# An integrative machine learning framework for classifying SEER breast cancer

**DOI:** 10.1038/s41598-023-32029-1

**Published:** 2023-04-01

**Authors:** P. Manikandan, U. Durga, C. Ponnuraja

**Affiliations:** 1 Department of Data Science, Loyola College, Chennai, 600 034 India; 2 ICMR-National Institute for Research in Tuberculosis, Chennai, 600 031 India

**Keywords:** Breast cancer, Machine learning

## Abstract

Breast cancer is the commonest type of cancer in women worldwide and the leading cause of mortality for females. The aim of this research is to classify the alive and death status of breast cancer patients using the Surveillance, Epidemiology, and End Results dataset. Due to its capacity to handle enormous data sets systematically, machine learning and deep learning has been widely employed in biomedical research to answer diverse classification difficulties. Pre-processing the data enables its visualization and analysis for use in making important decisions. This research presents a feasible machine learning-based approach for categorizing SEER breast cancer dataset. Moreover, a two-step feature selection method based on Variance Threshold and Principal Component Analysis was employed to select the features from the SEER breast cancer dataset. After selecting the features, the classification of the breast cancer dataset is carried out using Supervised and Ensemble learning techniques such as Ada Boosting, XG Boosting, Gradient Boosting, Naive Bayes and Decision Tree. Utilizing the train-test split and k-fold cross-validation approaches, the performance of various machine learning algorithms is examined. The accuracy of Decision Tree for both train-test split and cross validation achieved as 98%. In this study, it is observed that the Decision Tree algorithm outperforms other supervised and ensemble learning approaches for the SEER Breast Cancer dataset.

## Introduction

According to factsheets by World Health Organization (WHO), breast cancer is the second foremost root of cancer death in women and it has a high mortality rate^1^. Breast cancer disease is a disorder in which the cells in the breast raise out of control. The Breast cancer manifests itself in a diversity of ways. Breast cancer type is resolute by which cells in the breast developed as cancerous. About ninety percentage of breast cancer disease are caused by genetic abnormalities that happen as an effect of the ageing process, and 5–10% of breast cancers are caused by an irregularity that is hereditary from the parents. Modern medical diagnoses are based on information gathered through clinical remark or other trials. Several researchers have emphasized the importance of Artificial Intelligence and Machine Learning in healthcare domains^2^. Correlation analysis and Principal Component Analysis (PCA) are used for the purpose of dimensionality reduction and to make the models perform well^3^. Supervised and Unsupervised learning methods are used for the Detection of Breast Cancer through Clinical Data^4^. Crystall algorithm is used to select the important features for the prediction of survival time for Breast Cancer Patients^5^. A combination of scaling and Principal Component Analysis (PCA) are used for feature selection in the breast tumor dataset. Both the supervised and unsupervised machine learning models are used for classifying the breast cancer dataset^6^. Minimal Redundancy Maximal Relevance and Chi-Square Algorithms are used to select the features from the breast cancer dataset^7^. Various feature selection and classification techniques based on Deep Learning have been assessed in the existing literature^8^. The main goal of this research work is to categorize and predict the alive and death status of cancer patients. The remaining section of this manuscript is prepared as follows- Section "Literature review" defines the Literature Review, Section "Materials and methods" describes the Materials and Methodology and Section "Results and discussion" illustrates the experimental results on the SEER breast cancer dataset and discusses the outcomes. Lastly, the conclusion and future enrichment are specified in Section "Conclusion and future enhancement".

## Literature review

Feature selection techniques such as Recursive Feature Elimination, Forward Feature Selection, f-test and correlation are used with Wisconsin breast cancer data for extraction of important features^9^. Principal Component Analysis technique was used to indicate the genomic variants in rare genetic diseases^10^. Chi-Square, Singular Vector Decomposition and PCA are used to select the features from the breast cancer dataset^11^. PCA was used to extract the features from the Surface Enhanced Raman spectroscopy (SERS) and Raman Spectroscopy (RS) breast cancer serum^12^. Exploratory Data Analysis (EDA) of the breast cancer dataset was performed using PCA technique^13^. Receiver operating characteristic curve (ROC) and PCA method was used to visualize the prediction ability of various methods^14^. Random Forest and Principal Component Analysis methods are combined for attribute selection and accurate diagnosis of breast cancer patients^15^. Recent literatures for classifying breast cancer dataset have also been reviewed. Artificial Intelligence techniques such as Machine Learning and Deep Learning algorithms are used to perform the classification of breast cancer datasets^16^. Support Vector Machine (SVM) technique is employed for the classification of the Wisconsin breast cancer dataset^17^. An Improved Instance-Based K-Nearest Neighbour (IIBK) Classification was developed for solving the problem of Imbalanced Datasets with Enhanced Preprocessing^18^. Random Forest, KNN (k-Nearest-Neighbor) and Naive Bayes model are also used for the classification of the Wisconsin dataset^19^. MicroRNA regulated protein interaction pathways is predicted using fuzzy-based algorithms and also to rank Arabidopsis Thaliana^20^. SVM as well as K-Nearest Neighbor (KNN) algorithms are used to perform breast cancer prediction using tenfold cross-validation^21^. Four machine learning models such as Decision Tree, KNN, Binary SVM and AdaBoost are used to predict the stages of cancer^22^.

The time complexity of Naïve Bayes, logistic regression and decision tree is analysed using the breast cancer dataset. Logistic regression performs better than the other classifiers with the highest accuracy^23^. The dynamic ensemble learning algorithm is used to automatically identify the number of neural networks and their architecture^24^. The Bacterial Foraging Optimization—Genetic Algorithm (BFO-GA) is developed for solving the problem of Multiple Sequence Alignment (MSA)^25^. Support Vector Machine, Random Forest and Bayesian Networks are used to classify the Wisconsin dataset^26^. Enhanced Artificial Neural Network is used for predicting Protein Fold Recognition and Structural Class Prediction^27^. Protein sequence prediction and analysis are performed using a hybrid Knuth-Morris Pratt (KMP) and Boyer-Moore (BM) method^28^. Decision Tree based model evaluation is performed for breast cancer dataset using data mining approaches^29^. The Particle Swarm Optimization (PSO) algorithm was used to identify the cancer specific gene selection^30^. Deep Convolution Neural Networks with multi scale kernels is used to automate the diagnosis of breast ultrasonography images^31^. Convolutional Neural Network based diagnosis method was used to detect the early stage of breast cancer using image dataset^32^. An Improved Convolution Neural Network was developed to classify the brain tumors using Magnetic Resonance Image (MRI) data^33^. There are various metrics to evaluate the machine learning models. Accuracy, precision and recall are used to evaluate the models such as Logistic Regression, Nearest Neighbor and Support Vector Machines^34^. Propensity score matching was used to compare the survival outcomes in breast cancer patients, based on the axillary surgery^35^. The global burden of breast cancer in 2020 and the burden breast cancer in the year of 2040 was predicted^36^. Methods based on machine learning can assist physicians in reducing the number of false positive and false negative decisions. Based on the existing literatures, this research work focused on classifying the SEER breast cancer dataset using Machine Learning models such as Supervised and Ensemble Learning. In the exiting literature^29^, the features were chosen according to previously published sources and the features were chosen at random that were influenced by clinical and statistical significance. The current work focuses primarily on the features that were chosen from the SEER dataset using advanced feature selection techniques like Variance Threshold and PCA methods. These features were strongly correlated with the features chosen at random in the earlier work. All machine learning algorithms that performed the classification used the chosen features as input.

## Materials and methods

### Dataset description

**Figure 1:**
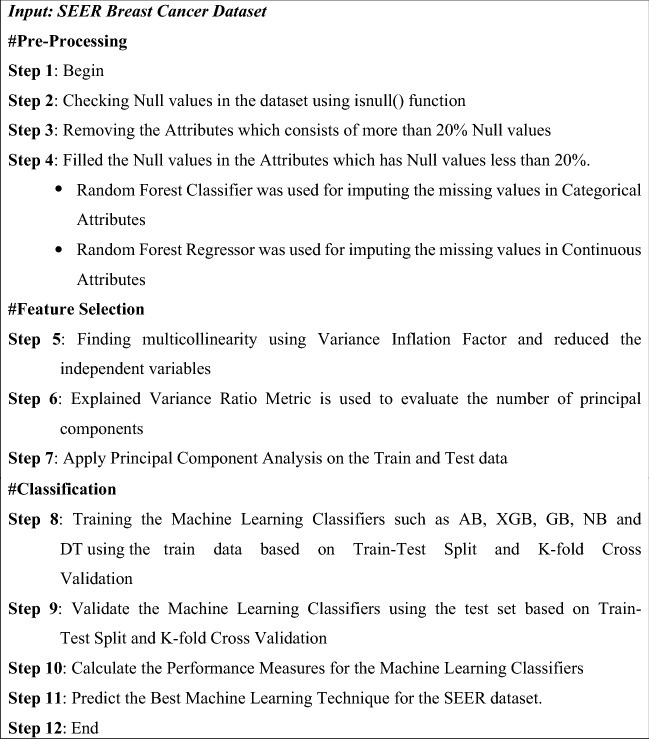
Pseudocode for the proposed classification framework.

**Figure 2:**
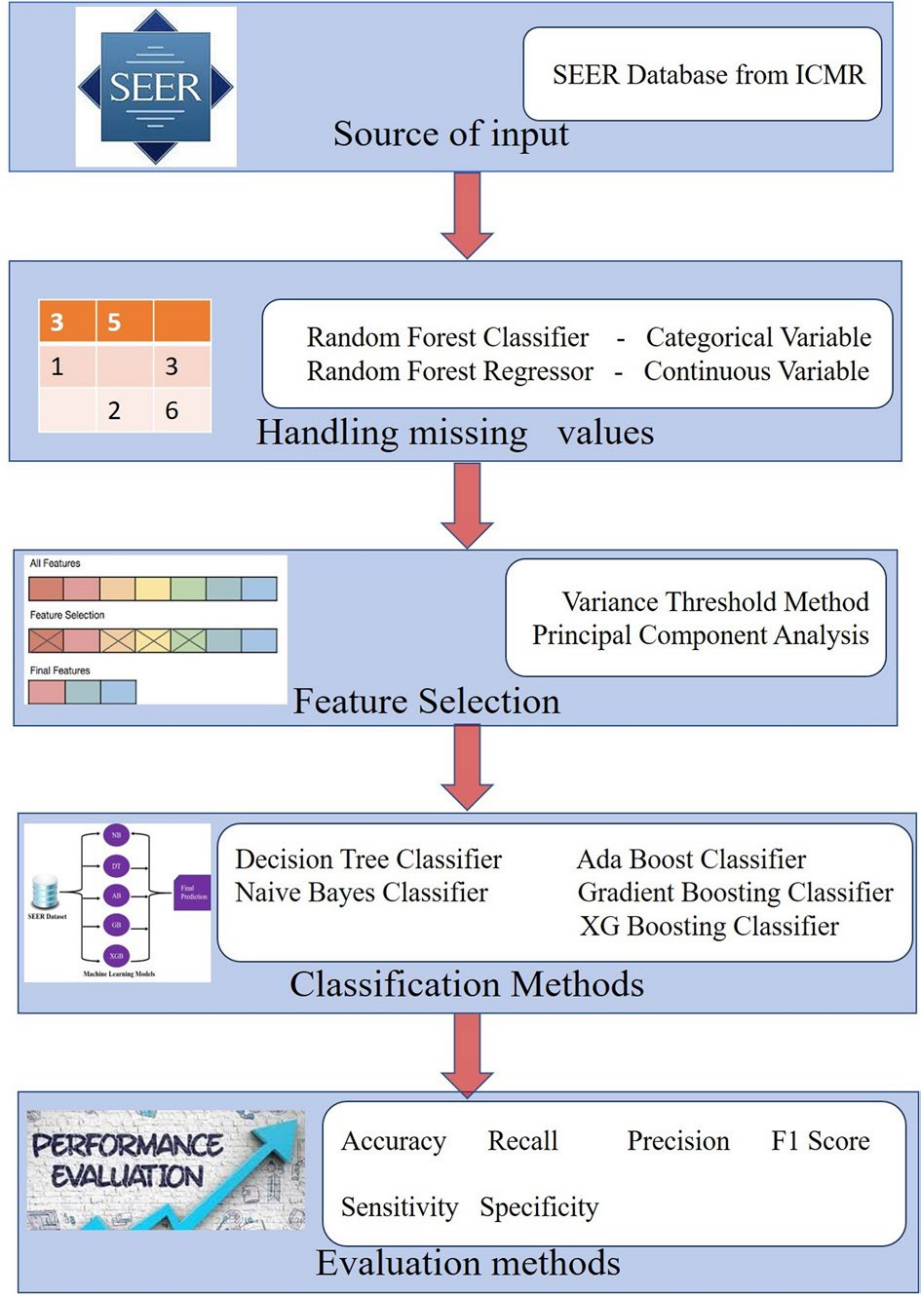
Overall system architecture for this research work.

Cancer incidence data for all types of cancer can be found in the Surveillance, Epidemiology, and End Results (SEER) database (1972–2012). The SEER dataset consists of 7,12,319 breast cancer patient records with 149 features and this database^37^ is sustained by the National Cancer Institute (NCI) that comprises data on cancer incidence, prevalence, survival, and mortality in the United States. It was created by the United States government to collect data on cancer patients across the country. By law, all hospitals, clinics, laboratories, surgery sections, and organizations involved in the diagnosis and treatment of cancer must report information to this institute, which will be reviewed before being entered into the SEER database. The pseudocode for the proposed classification framework is shown in Fig. 1 and the overall architecture for this research work is shown in Fig. 2.

### Handling missing values

The dataset contains more missing values. Hence, the features which have missing values of more than 20% are removed. The categorical features are imputed using the Random Forest classifier and continuous features are imputed using Random Forest Regressor. The parameter for the Random Forest Classifier technique is configured as the number of estimators is set to 100, criterion is set to *gini* with bootstrapping. The parameter for the Random Forest Regressor technique is configured as the number of estimators is set to 100, criterion is set to *squared_error* with bootstrapping.

### Feature selection

Feature selection aims to discover the finest set of features that can be used to build models for the phenomena being studied. Because it is very hard to use more features and it may cause overfitting. In this research, a few feature selection techniques such as Variance Threshold and Principal Component Analysis (PCA) have been used to improve the model performance.

#### Variance threshold

For feature selection, the variance threshold method is applied. It eliminates all attributes with variances below a predetermined level. By default, it removes all attributes with zero variance, or attributes having the same value across all instances. The relationship between features and the target variable is ignored by the variance threshold. A simple baseline method called Variance Threshold (VR) eliminates all features with zero variance. Nine features in the SEER dataset show too little variation (less than or equal to 0%), according to the variance threshold technique. We currently have 50 features. Table 1 displays the significant risk factors from the SEER breast cancer dataset.

**Table 1:** Important risk features of breast cancer disease in SEER dataset.

Variable	Levels	Frequency
Sex	Male	4641
	Female	707,678
Age	14–30	43
	31–40	217
	41–50	742
	51–60	2826
	61–70	5804
	71–80	6467
	80 and above	3901
Origin	0	681,921
	1	2901
	2	1374
	3	237
	4	1759
	5	2259
	6	13,947
	7	4207
	8	80
FIRSTPRM	0	187,690
	1	524,629
Status	0	385,446
	4	326,873

#### Principal component analysis (PCA)

The Principal Component Technique was used to solve the problem of multicollinearity and the number of principal components was discovered using Variance Inflation Factor (VIF). This model used 13 components out of a total of 50.

### Methodology

#### Decision tree classifier

Decision tree classifier is used to choose whether to split a node into two or more sub-nodes. For constructing decision trees, we can employ a diversity of machine learning models. The similarity of the resultant sub-nodes enhances with the creation of sub-nodes. The purity of the node expands as the target variable is increased. The decision tree splits the nodes into sub-nodes based on the input features, then selects the split that produces the maximum similar sub-nodes. This technique tries to divide the input dataset into the smallest subset possible at each split. The aim of Decision Tree algorithm is to reduce the loss metric value as much as possible. The loss functions such as Gini Impurity and Entropy are used to collate the class distribution beforehand and after the split. The loss metric named Gini Impurity is used to measure the variation between different classes. The parameter for the Decision Tree method is configured as the criterion is set to *gini*, splitter as best, minimum sample split as 2 and minimum sample leaf as 1.

#### Naive Bayes (NB) classifier

This Naïve Bayes model has newly gained popularity and is being used more frequently. It’s a statistical pattern recognition technique that makes a reasonable assumption about how data is generated. The parameters of NB are estimated using training samples in this model. This is a simple classifier, based on the assumption that all sample attributes are independent. Once the hypothesis is false, Naïve Bayes classifies the data in a perfect manner, because the classification hypothesis is only a symbol of function approximation, and the function estimate is achieved with low accuracy, whereas the classifier's accuracy is high. The parameter for the Naïve Bayes method is configured with the var smoothing as 1e-9. The conditional probability of individual variable X_k_ assumed the class label C is learned by Nave Bayes using training data and the conditional probability of individual variable X_k_ is specified the class label C. The Bayes rule is used to calculate the probability of C specified a particular instance, ${X}_{1}$,…${X}_{n}$, using Eq. (1):

1 $$ {\text{P}}({\text{C}} = {\text{c}}|X_{1} = x_{1} , \ldots ,X_{n} = x_{n} ) $$

Because this classifier is based on the hypothesis that variables are conditionally independent. Equation (2) is used to calculate the posterior probability of the class:

2 $$ {\text{P}}({\text{C}} = {\text{c}}|X_{1} = x_{1} , \ldots ,X_{n} = x_{n} ) = {\text{P}}({\text{C}} = {\text{c}})*\Pi {\text{Xk}}\,{\text{P}}(X_{k} = x_{k} |{\text{C}} = {\text{c}}) $$

The class with the highest posterior probability Eq. (3) is the classification result.

3 $$ max_{c} \Pi {\text{X}}_{{\text{k}}} {\text{P}}(X_{k} = x_{k} |{\text{C}} = {\text{c}}) $$

#### AdaBoost (AB) classifier

Freund and Schapire invented the adaptive boosting machine learning algorithm^38^, which is abbreviated as AB. AB is a meta-algorithm that works in aggregation with other learning algorithms to enhance the performance. AdaBoost is a training method for boosted classifiers, which are classifiers that have the form Eq. (4):

4 $$ FT = \sum_{t = 1}^{T} = 1^{f} t({\text{x}}), $$

where individual f_t_ is a poor learner that receipts input and yields a real-valued outcome that indicates the sample's class. The predicted sample class is identified by the weak learner outcome, and the value designates the level of sureness in that classification. Likewise, if the data is thought to be in a positive class, the T-layer classifier will be positive, else it will be negative. For each sample in the training set, individual weak learner model produces an output, hypothesis h(x_i_). Weak learner is elected and assumed a coefficient at respective iteration, *t*, so that the sum training error of the resulting t-stage boost classifier is minimized (Eq. (5)).

5 $$ E_{t} = \Sigma_{i} {\text{E}}\left[ {{\text{F}}_{{\text{t}}} - 1(x_{i} ) + \alpha_{t} h(x_{i} )} \right] $$

F_t_ − 1(x_i_) denotes the boosted classifier, E(F) denotes error function, and ${f}_{t}$(x) =${ \alpha }_{t}h$ (${x}_{i}$) denotes the weak learner for inclusion in the final classifier. In Adaboost, each new stage's classification is built on samples that have been incorrectly classified. Although AB is sensitive to noise and outliers data and it outperforms other learning algorithms in terms of overfitting. Random classification is the algorithm's base classifier (50 percent). The parameter for the AdaBoost method is configured as the number of estimators is set to 50, estimator is set to *none*, learning rate as 1.0 and the SAMME.R algorithm is used.

#### XG boost classifier

XGBoost (XGB) is classified as a boosting technique in Ensemble Learning. To improve prediction accuracy, ensemble learning combines multiple models into a collection of predictors. In the boosting technique, previous models' errors are attempted to be corrected by subsequent models by adding weights to the models. Gradient Boosted algorithms, unlike other boosting algorithms, optimise the loss function rather than increasing the weights of misclassified branches. With some regularisation factors, XGBoost is a more advanced gradient boosting implementation. The parameter for the XGBoost method is configured as the verbosity is set to 1 and the gbtree is used as booster.

#### Gradient boosting algorithm

Gradient boosting (GB) is a boosting algorithm based on the ensemble techniques. In this model, each predictor alters the error of the previous model. The training sample weights are not adjusted in Adaboost. As an alternative, each model is trained using the ancestor's residual errors as labels. Gradient Boosting technique use CART (Classification and Regression Trees) as the base learner. The Gradient Boosting is an ensemble model that can be made up of N number of trees. The first tree model is trained using the feature matrix X and labels y. The residual errors (r1) in the first tree training set are considered using the predictions labelled y1 (hat). The second tree is trained using Tree1's feature matrix X and residual errors r1 as labels. Using the predicted results r1, the residual r2 is calculated (hat). This procedure is repetitive until all N trees in the ensemble have been trained. The parameter for the Gradient Boosting method is configured as the number of estimators is set to 100, criterion is set to *friedman_mse*, the learning rate as 0.1 and log loss is used as loss metric.

Shrinkage occurs when the prediction of each model in the ensemble is grown by the learning rate (lr), which ranges from 0 to 1. All the trees have been trained and each tree predicts a label with Eq. (6) providing the ultimate prediction. The mathematical notations which are used in this research work in shown in Supplementary Table S1.

6 $$ {\text{y}}\left( {{\text{pred}}} \right) = {\text{y1}} + \left( {{\text{lr}}*{\text{ r1}}} \right) + \left( {{\text{lr }}*{\text{r2}}} \right) + \cdots + \, \left( {{\text{lr }}*{\text{ rN}}} \right) $$

## Results and discussion

Machine learning models that are supervised and ensemble predict breast cancer survival. The proposed method to predict breast cancer survival included five machine learning models, including NB, Decision tree classifier, Ada Boost, XG Boost, and Gradient Boosting classifier. The experiments are performed using an Intel(R) Core (TM) i5-1235U 1.30 GHz CPU with 8 GB of RAM and Windows 11 as the operating system. Python 3.8 was used to develop the proposed framework.

### Performance metrics

The Performance metrics which are used in this research work are discussed below.

#### Accuracy

Accuracy refers to the correctly classified instances by the total amount of instances present in the SEER dataset (Eq. 7).

7 $$\mathrm{Accuracy}=\frac{\mathrm{TP}+\mathrm{TN}}{\mathrm{TP}+\mathrm{FP}+\mathrm{TN}+\mathrm{FN}}$$

where TP = True Positive, FP = False Positive, TN = True Negative, FN = False Negative, TP = Dead persons correctly known as dead. TN = Alive persons correctly recognized as dead. FP = Alive persons wrongly recognized as dead. FN = Dead persons wrongly recognized as alive.

#### TP rate

It is used to find the high true-positive rate using the Eq. (8). The true-positive rate is also known as sensitivity and it measures the part of actual positives which are appropriately recognized.

8 $$\mathrm{TPR}=\frac{\mathrm{TP}}{\mathrm{TP}+\mathrm{FN}}$$

#### FP rate

The False Positive rate (Eq. 9) refers to the probability of falsely refusing the null hypothesis for a specific test. It usually refers to the anticipation of the false positive ratio.

9 $$\mathrm{FPR}=\frac{\mathrm{FP}}{\mathrm{FP}+\mathrm{TN}}$$

#### F-measure

F-Measure is the mixture of both precision and recall (Eq. 10), which is used to calculate the score. This kind of measure is often used in the field of Information Retrieval to estimate the query classification performance.

10 $$\mathrm{F}-\mathrm{ Measure }= 2*\frac{\mathrm{Recall}*\mathrm{ Precision }}{\mathrm{Recall}+\mathrm{Precision}}$$

where, Precision = $\frac{\mathrm{TP}}{\mathrm{TP}+\mathrm{FP}}$ and Recall = $\frac{\mathrm{TP}}{\mathrm{TP}+\mathrm{FN}}$

### Performance of the proposed model

The SEER breast cancer data contains 149 features with 712,319 records. In the SEER data, six categorical features such as 'siteo2v', 'eod13', 'eod2', 'icdot10v', 'plc_brth_cntry' and 'plc_brth_state' which will not contribute to the model as we want. Hence, the six features are dropped. Then we found that the SEER data has some features which have more null values. Around 84 features have null values of more than 20%. Even if we try to impute them, it may impact the model in a bad way. So, we dropped those features as well. Now we are left with 58 features. Among 58 features we have 13 features that have null values of less than 20% (Table 2) and 45 features which don’t have null values.

**Table 2:** Features having null values in SEER breast cancer dataset.

Attributes	No. of missing values
MAR_STAT	28,029
RACE	2193
ORIGIN	3634
AGE_DX	39
SEQ_NUM	21
DX_CONF	4290
RAD_SURG	645
AGE_REC	39
ICCC3WHO	108,423
ICCC3XWHO	108,423
RAC_RECA	2789
RAC_RECY	2789
IHS	134,659

The missing values are imputed using Random Forest Classifier for categorical features and Random Forest Regressor for continuous features. After imputing the missing values, the important features are selected using the Variance Threshold method. By using this method, 50 features are selected among 58 features. For finding the multicollinearity, the Variance Inflation Factor (VIF) value is calculated for the 50 features and it is shown in Table 3.

**Table 3:** Variance inflation factor (VIF) value for the 50 features.

Id	Variables	VIF
0	MAR_STAT	6.129996
1	RACE	1.281009
2	ORIGIN	3.796222
3	NHIA	9.360221
4	SEX	968.1161
5	AGE_DX	1550.369
6	SEQ_NUM	17.29868
7	DATE_mo	4.600695
8	DATE_yr	30.39801
9	LATERAL	5.766537
10	HISTO2V	189,864.8
11	BEHO2V	88,968.06
12	HISTO3V	288,649
13	BEHO3V	Infinity
14	GRADE	5.0837
15	DX_CONF	5.746266
16	REPT_SRC	4.085283
17	NO_SURG	1.678718
18	RADIATN	1.435221
19	RAD_SURG	1.897931
20	REC_NO	45.64985
21	TYPEFUP	160.756
22	AGE_REC	1708.033
23	ICDOTO9V	890.8556
24	ICCC3WHO	384,864.2
25	ICCC3XWHO	96,881.94
26	BEHANAL	infinity
27	HISTREC	430.9573
28	RAC_RECA	149.9788
29	RAC_RECY	94.15077
30	NHIAREC	8.602672
31	HST_STGA	3.559645
32	NUMPRIMS	26.27406
33	FIRSTPRM	32.60563
34	STCOUNTY	3.531148
35	ICD_5DIG	146,226.7
36	CODKM	146,228.1
37	IHS	1.10791
38	AYA_RECODE	1815.535
39	DTH_CLASS	51.3407
40	O_DTH_CLASS	56.58297
41	INTPRIM	527.1385
42	ERSTATUS	162.7737
43	PRSTATUS	169.1439
44	SRV_TIME_MON	85.90525
45	SRV_TIME_MON_FLAG	927.3435
46	SRV_TIME_MON_PA	88.26923
47	SRV_TIME_MON_FLAG_PA	931.0642
48	HER2	3067.783
49	BRST_SUB	4235.518

After finding the VIF values, the dataset is performed with the Standard Scaler method and then it is split into training and testing records. The Xtrain consists of 498,623 records with 50 features and Xtest consists of 213,696 records with 50 features. To solve the problem of multicollinearity, the Principal Component Analysis (PCA) dimensionality reduction technique is used to reduce the feature dimensions. For achieving this, the Principal Explained Variance Ratio method is used to find the number of components. Now the features end up with 13 components and the Principal Explained Variance Ratio for the 13 features is shown in Table 4.

**Table 4:** Principal explained variance ratio for the 13 features generated by principal component analysis (PCA) algorithm.

S. no	Principal explained variance ratio
1	0.17275442
2	0.09822647
3	0.0905788
4	0.07328113
5	0.05573796
6	0.05357803
7	0.04697576
8	0.03966092
9	0.03453351
10	0.02726182
11	0.02418573
12	0.02144122
13	0.02121837

In this study, five machine learning algorithms are used to predict the survival of breast cancer such as Naïve Bayes, Decision tree classifier, Ada Boost, XG Boost, and Gradient Boosting classifier. In the Decision Tree, the criterion for determining the quality of a split is entropy, which is calculated using information gain given by entropy, and the random state is 0 for generating random states. When building an NB classifier with zero training instances, the default precision for numeric attributes is 0.1. In Adaboost, the Decision Stump algorithm is chosen as the base classifier. The number of iterations to be accomplished is set to 10 and the weight pruning threshold is set to 100. In the Gradient Boosting Classifier log loss function was used and the learning rate was set to 0.1, the criterion is friedman_mse. In the XG Boost classifier gbtree booster was used and the learning rate is 0.3. These machine learning models have been implemented, and the comparison results are summarized in Tables 5 and 6. The alive and death count of breast cancer patients predicted by machine learning models is shown in Table 7. The comparison of machine learning models (percent) by train test split and cross-validation strategy, including NB, Decision tree classifier, Ada Boost, XG Boost, and Gradient Boosting classifier is shown in Tables 5 and 6.

**Table 5:** Comparison of performance metrics for supervised and ensemble learning methods using train test split method.

Algorithms	Accuracy	Precision	Recall	F1 Score	TP	FP	Sensitivity	Specificity
NB	92	0.90	0.96	0.93	0.96	0.11	0.96	0.88
**DT**	**98**	**0.98**	**0.98**	**0.98**	**0.98**	**0.019**	**0.98**	**0.98**
AB	97	0.98	0.99	0.98	0.99	0.016	0.99	0.98
GB	90	0.89	0.94	0.92	0.94	0.074	0.94	0.92
XGB	90	0.90	0.93	0.91	0.93	0.080	0.93	0.92

Significant values are given in bold.

**Table 6:** Comparison of performance metrics for supervised and ensemble learning methods using a fivefold cross-validation method.

Algorithms	Accuracy	Precision	Recall	F1 Score	TP	FP	Sensitivity	Specificity
NB	93	0.94	0.92	0.92	0.92	0.13	0.92	0.89
**DT**	**98**	**0.99**	**0.97**	**0.97**	**0.97**	**0.02**	**0.97**	**0.97**
AB	97	0.96	0.98	0.96	0.98	0.019	0.98	0.96
GB	89	0.92	0.86	0.88	0.86	0.070	0.86	0.93
XGB	91	0.89	0.93	0.90	0.93	0.080	0.93	0.93

Significant values are given in bold.

**Table 7:** Alive and death count of breast cancer patients predicted by machine learning techniques.

Algorithms	Alive	Death
NB	409,441	302,878
**DT**	**373,879**	**338,440**
AB	359,017	353,302
GB	402,893	309,426
XGB	399,049	313,270

Significant values are given in bold.

**Figure 3:**
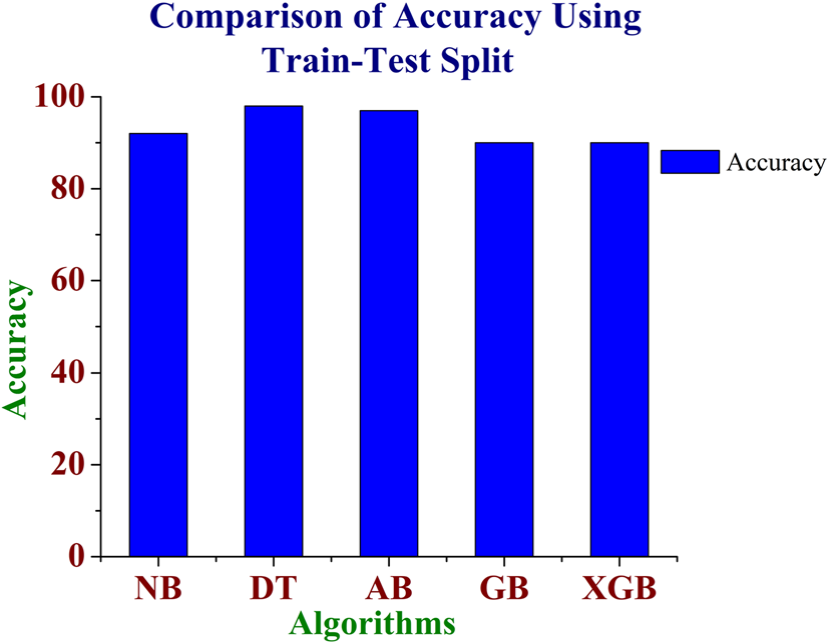
Comparison of accuracy for the various machine learning models using train- test split method.

**Figure 4:**
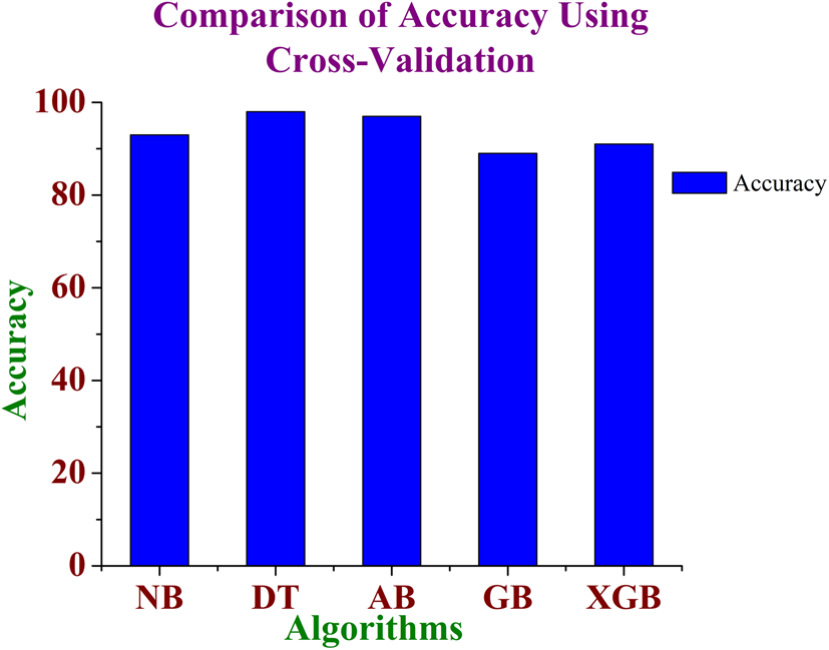
Comparison of accuracy for the various machine learning models using cross-validation method.

**Figure 5:**
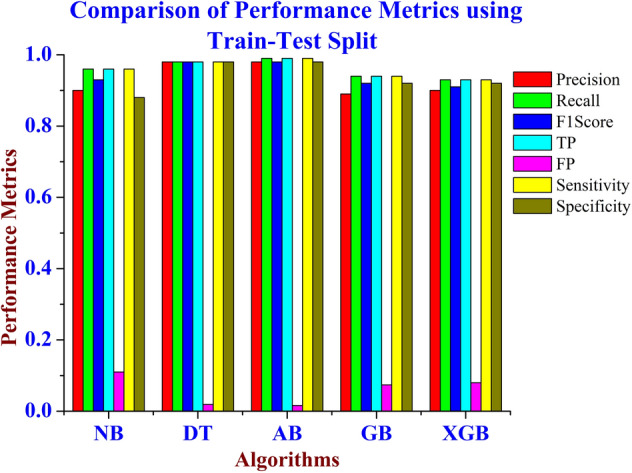
Comparison of performance metrics for the various machine learning techniques using the train-test split method.

**Figure 6:**
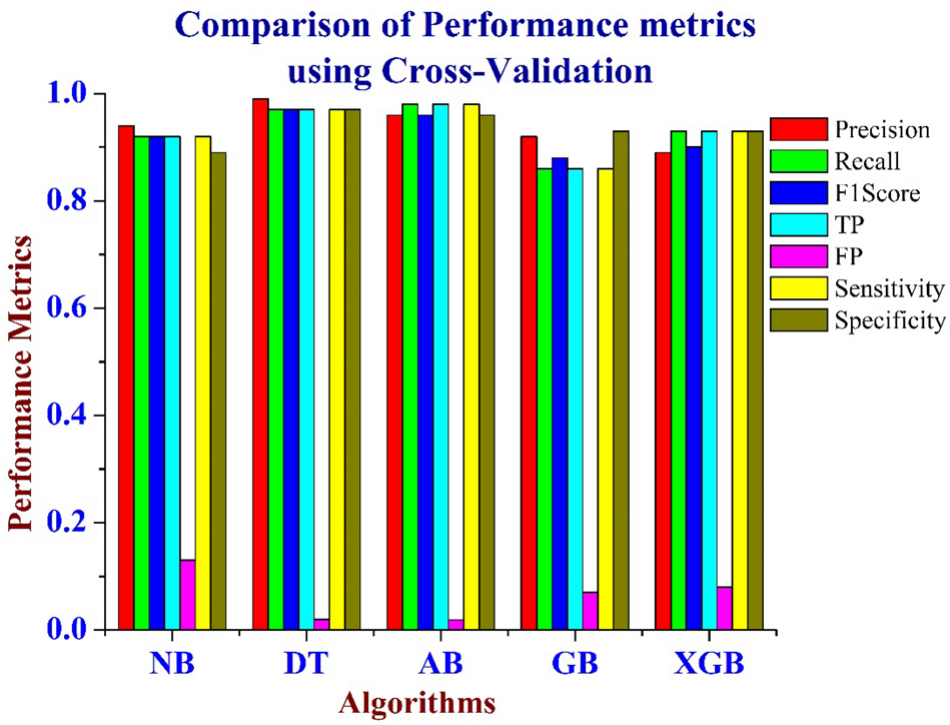
Comparison of performance metrics for the various machine learning techniques using the cross-validation method.

Figures 3 and 4 shows the comparison of Accuracy for the various machine learning techniques such as Naïve Bayes, AdaBoost, Decision Tree, Gradient Boosting and XG Boosting algorithms using Train-Test Split and Cross Validation Methods. From Figs. 3 and 4, it is inferred that the Decision Tree algorithm performs better than the other algorithms in terms of Accuracy. Figure 5 shows the comparison of performance metrics values for the various machine learning algorithms using the Train-Test Split method. From Fig. 5, it is inferred that the Decision Tree algorithm provides better results compared to other machine learning models. The Fig. 6, shows the comparison of performance metrics values for the various machine learning algorithms using the Cross-Validation method. From Fig. 6, it is inferred that the Decision Tree algorithm provides better results compared to other machine learning algorithms.

These machine learning models are associated in terms of precision, recall, F1 score, and accuracy using train test split and cross-validation strategies. From the experimental results, it is inferred that the decision tree model achieved 98% accuracy which is the highest among those other machine learning models. For the SEER breast cancer dataset, it is inferred that the Decision Tree classifier algorithm performs 6.12% better than the NB algorithm, 1.02% better than the Adaboost algorithm and 8.16% better than the GB and XGB algorithms using the train test method. For the cross-validation method, it is inferred that the Decision Tree classifier algorithm performs 5.1% better than the NB algorithm, 1.02% better than the Adaboost algorithm, 9.18% better than the GB and 7.14% better than the XGB algorithm. From the experimental results it is inferred that the Decision Tree outperforms the other machine learning models. As shown in Tables 5 and 6, the Decision Tree machine learning model is the best model for classifying the SEER breast cancer disease dataset.

## Conclusion and future enhancement

Given that breast cancer is one of the most common causes of death for women, early detection is crucial. The burden on doctors can be decreased by using automatic classification systems as diagnostic tools. Modern machine learning classifiers make it possible to identify breast cancer tumours early. Even while false positive and false negative results are frequently acknowledged to be significant in medical research, the majority of past studies have primarily focused on accuracy. As a result, we looked at various performance metrics in addition to accuracy, precision, and recall. In this work, variance threshold and principal component analysis were used to determine the features. Then, the chosen features are fed into the machine learning classifiers as input to carry out the classification task. This study evaluates the effectiveness of different machine learning classification methods for predicting breast cancer survival, including Naive Bayes, Decision Tree, Ada Boost, XG Boost, and Gradient Boosting classifiers. The decision tree approach was the most successful, according to the comparative results. In the future, several machine-learning techniques might be used to classify datasets pertaining to the breast cancer disease.

## Supplementary Information

Supplementary Information.

## Data Availability

The datasets used and/or analysed during the current study are available from the corresponding author upon reasonable request.
