# Supplementary material for: An integrative machine learning framework for classifying SEER breast cancer
Source: Sci Rep. 2023 Apr 1;13:5362. doi: 10.1038/s41598-023-32029-1 (PMC10067827; doi:10.1038/s41598-023-32029-1)
Supplement: Supplementary file 1 — Supplementary Information. [file 41598_2023_32029_MOESM1_ESM.docx]

##### **An Integrative Machine Learning Framework for Classifying SEER Breast Cancer**

*^*^Manikandan P^1^, Durga U^2^, ^*^Ponnuraja C ^3^*

*^1^Assistant Professor, ^2^Student, ^3^Scientist E*

*^1,2^Department of Data Science, Loyola College, Chennai – 600 034, India.*

*^3^ICMR - National Institute for Research in Tuberculosis, Chennai, 600 031*

[*manimkn89@gmail.com^1^*](mailto:manimkn89@gmail.com1), [*20pds020@loyolacollege.edu*](mailto:20pds020@loyolacollege.edu) *^2^,* [*cponnuraja@gmail.com*](mailto:cponnuraja@gmail.com)*^3^*

** - Corresponding authors*

TABLE 1: Mathematical symbols and notations are used in this research work.

| Symbol | Description |
| --- | --- |
| X_k_ | conditional probability of individual variable |
| C | Class Label |
| $X_{1}$,...$X_{n}$ | Instances |
| P | Probability |
| f_t_ | Poor learner |
| F_t_-1(x_i_) | Boosted classifier |
| E(F) | Error function |
| $f_{t}$(x) =$\alpha_{t}h$ ($x_{i}$) | Weak Learner |
| X | Feature matrix |
| Y | Labels |
| r1 | Residual errors |
| N | Number of Trees |
| lr | Learning rate |
| TP | True Positive |
| FP | False Positive |
| TN | True Negative |
| FN | False Negative |
